# Socioeconomic inequalities in effectiveness of and compliance to workplace health promotion programs: an individual participant data (IPD) meta-analysis

**DOI:** 10.1186/s12966-020-01002-w

**Published:** 2020-09-04

**Authors:** Pieter Coenen, Suzan J. W. Robroek, Allard J. van der Beek, Cécile R. L. Boot, Frank J. van Lenthe, Alex Burdorf, Karen M. Oude Hengel

**Affiliations:** 1 Department of Public and Occupational Health, Amsterdam UMC, Vrije Universiteit Amsterdam, Amsterdam Public Health Research Institute, Van der Boechorststraat 7, 1081 BT Amsterdam, The Netherlands; 2 Department of Public Health, Erasmus University Medical Center, P.O. Box 2040, 3000 CA Rotterdam, The Netherlands; 3 Department of Work, Health and Technology, Netherlands Organisation for Applied Scientific Research TNO, Schipholweg 79-86, 2316 Leiden, The Netherlands

**Keywords:** Healthy lifestyle, Risk reduction behavior, Meta analysis, Workplace

## Abstract

**Background:**

This individual patient data (IPD) meta-analysis aimed to investigate socioeconomic inequalities in effectiveness on healthy behavior of, and compliance to, workplace health promotion programs.

**Methods:**

Dutch (randomized) controlled trials were identified and original IPD were retrieved and harmonized. A two-stage meta-analysis was conducted where linear mixed models were performed per study (stage 1), after which individual study effects were pooled (stage 2). All models were adjusted for baseline values of the outcomes, age and gender. Intervention effects were assessed on physical activity, diet, alcohol use, and smoking. Also, we assessed whether effects differed between participants with low and high program compliance and. All analyses were stratified by socioeconomic position.

**Results:**

Data from 15 studies (*n* = 8709) were harmonized. Except for fruit intake (beta: 0·12 [95% CI 0·08 0·15]), no effects were found on health behaviors, nor did these effects differ across socioeconomic groups. Only participants with high compliance showed significant improvements in vigorous and moderate-to-vigorous physical activity, and in more fruit and less snack intake. There were no differences in compliance across socioeconomic groups.

**Conclusions:**

Workplace health promotion programs were in general not effective. Neither effectiveness nor compliance differed across socioeconomic groups (operationalized by educational level). Even though stronger effects on health behavior were found for participations with high compliance, effects remained small. The results of the current study emphasize the need for new directions in health promotion programs to improve healthy behavior among workers, in particular for those in lower socioeconomic position.

## Introduction

Robust patterns of socioeconomic inequalities in mortality remain a key challenge in public health [1]. Unhealthy behaviors (smoking, poor diet and physical inactivity) contribute substantially to these inequalities [2, 3]. Given that health behaviors are potentially modifiable, they are good entry points for the reduction of health inequalities. However, still little is known regarding how to improve health behavior in lower socioeconomic groups.

The workplace is a good setting to deliver health promotion programs, because a substantial amount of daily time is spent at work, large groups of participants can be reached, and both environmental and individual-level interventions can be implemented [4]. Previous systematic reviews on worksite health promotion programs have shown positive effects on smoking cessation [5] and reducing alcohol intake [6]. Positive but small effects were found regarding improvement in diet [6–8]. Inconclusive effects were found on physical activity [6–10]. Despite these in general positive effects on health behavior, a recently published trial on a worksite health promotion program did not show positive long-term health effects [11].

Larger effects among workers in high compared to low socioeconomic position (SEP) groups in health promotion programs, mainly in programs with educational components, were reported in a systematic review [12]. Both the initial participation and compliance in such programs is generally lower among workers with a lower SEP [12–14]. However, a systematic review on initial participation in workplace health promotion did not find a consistently lower participation among workers in lower SEP [15]. Thus, the evidence for health inequalities of health promotion programs is inconclusive and is limited to conventional reviews with pooled study-level data. Individual studies often focused on single SEP groups only [15] and/or lacked the statistical power to stratify their analyses by SEP [12].

Against this background, we aimed to investigate whether socioeconomic inequalities exist in a) the effectiveness of and b) compliance to worksite health promotion programs focusing on increasing physical activity, healthy dietary behavior, reducing alcohol use, and smoking cessation. To address these aims, individual participant data (IPD) of Dutch (randomized) controlled trials were used. In contrast to study-level data in conventional meta-analyses, IPD allows for testing of interaction, mediation or moderation; data can be analyzed in a way that goes beyond what could or has been done by original individual studies [16]. For instance, this can be done by identifying relevant subgroups and to test mechanistic pathways [17]. Moreover, an IPD meta-analysis has the benefit of a larger number of data points, facilitating more statistically powerful and sound conclusions based on careful evaluation of modelling assumptions. We only used Dutch data to obtain a homogeneous dataset, since in the occupational context in the Netherlands all employees have access to occupational health care through their employer, who is responsible for sickness benefits during the first two years of sickness absence.

## Methods

This IPD meta-analysis was executed according to our published [18] and registered protocol (PROSPERO; CRD42018099878). The PRISMA-IPD statement was used to report our findings [19]. The Medical Ethical Committee of Erasmus MC declared that the Medical Research Involving Human Subjects Act does not apply to our IPD meta-analysis (MEC-2018-1143).

### Identification and inclusion of eligible studies

A systematic search was conducted to identify Dutch studies with health promotion programs aimed at improving health behavior among workers [18]. Briefly, relevant published studies were identified via searches in electronic databases (Embase, Medline Ovid, Web of Science, Cochrane Central and Google Scholar) and snowball searches in reference lists of included articles. Unpublished studies were identified via screening trial registers, databases of major Dutch funding agencies and the Dutch database of health promotion programs. In the Netherlands, all employees have access to occupational health care through their employer, who is responsible for sickness benefits during the first two years of sickness absence. Due to this unique context, we will focus on trial data from the Netherlands only to derive at a homogeneous dataset.

Two independent reviewers screened all records for eligibility. In case of disagreement, consensus was reached in a meeting or by consulting a third reviewer. A total of 34 studies (with 88 articles) on health promotion programs, targeted at workers, conducted in the Netherlands, with an indicator of SEP, and with study designs of at least a pre- and post-program measurement and a reference control group were considered eligible [18].

A request to share the original data was sent to researchers of each eligible study. After agreeing with this request, the researchers were asked to sign a data transfer agreement and to transfer their data, code books and syntaxes. Data could be sent in various formats, and were checked for completeness, improbable values, consistency with original articles, and missing items. Researchers of the original studies were consulted in case of uncertainty on any of these issues. Moreover, researchers of the original studies gave their final approval for the current paper.

### Methodological quality and harmonization

Information on study design, program characteristics, characteristics of the participants, health behavior assessment, and methodological quality were extracted from the identified articles of each eligible study by one reviewer, and verified by another one. The extracted data were checked by the researcher(s) of the original studies.

Methodological quality was assessed using a modified version [20] of the Cochrane risk of bias tool [21], consisting of nine criteria regarding randomization, blinding, similarity of groups, compliance, loss to follow-up, intention-to-treat, confounder adjustment, data collection methods, and follow-up duration. On each item, a study could score positive if the quality criterion was met (1 point), negative if the criterion was not met (0 point), or unclear if the publication or additional information request by authors provided insufficient information (0 points). Summary scores were categorized excellent (8–9), good (5–7), fair (3–4), or poor (0–2).

All study data were harmonized using definitions of each of the variables as formulated by the research team, which are outlined in our study protocol [18] and are described in more detail in a code book. For some variables (e.g. physical activity outcomes) individual questionnaire items were used to estimate certain constructs, deviating from the construct reported in the original studies.

#### Health behavior

Each health behavior variable was harmonized at baseline and, if available, at two follow-up measurements to assess the immediate and sustained effects. Immediate was defined as the measurement directly following the health promotion program. Sustained was defined as the measurement after a follow-up period. As health promotion program duration and follow-up periods differed between studies, immediate and sustained effects had different definitions for each study.

As described in the codebook, physical activity was defined as moderate physical activity (MPA), vigorous physical activity (VPA) and moderate-to-vigorous physical activity (MVPA). Due to heterogeneity in physical activity definitions and assessment methods, outcomes were recoded into z-scores, using standard procedures per type of questionnaire.

Dietary behavior consisted of fruit, vegetable, snack and fat intake. Daily or weekly intake of each of these diet modalities was estimated and z-scores were calculated to accommodate for heterogeneity in definitions and assessment methods.

Alcohol intake was harmonized by expressing variables in units (e.g. glasses) of alcohol intake per week, which was, for the sake of consistency across outcome measures, also transformed into z-scores. Smoking was harmonized into a dichotomous variable with outcome categories ‘non-smoking’ (also including ex-smokers) and ‘smoking’.

#### Socioeconomic position

As we expected educational level to be the most frequently reported indicator of SEP, SEP was harmonized based on the 1997 International Standard Classification of Education (ISCED-97). SEP was defined as low (pre-primary, primary and lower secondary), intermediate (upper secondary), and high (post-secondary) education. In the Netherlands, educational level is considered to be a valid indicator for SEP, which is strongly associated with a large variety of health outcomes [22]. Nonetheless, a variable for education was lacking in one study with workers from a construction industry [23]. For that study, occupational class was used to define SEP, with blue collar workers being categorized as low SEP and the office workers as intermediate SEP.

#### Compliance

Compliance was defined as the adherence to the health promotion programs (e.g. number of sessions followed or number of consults received) expressed in a percentage. For multi-component health promotion programs an average of the compliance to the various program components was taken. Compliance was dichotomized per study to differentiate participants with low and high compliance, using a median split.

#### Additional variables

In accordance with the individual studies, age was considered as a continuous variable and gender as a dichotomous variable. In case of clustered trials, a variable indicating the clustering (e.g. worksite or company level) was composed.

### Statistical analysis

All individual datasets were merged into one IPD database. Data from participants within working age (18–67 years), with available data on SEP and relevant health behavior outcomes were included in further statistical analyses.

A two-stage meta-analysis approach was performed. In the first stage, IPD data of each study were analyzed separately using multi-level linear mixed models. In the second stage, the results per study were pooled in a meta-analysis using the Stata *admetan* function for each of the continuous outcome variables; i.e. physical activity (MPA, VPA and MPVA), diet (fruit, vegetable, snack and fat intake), and alcohol intake. Although smoking was harmonized as a dichotomous variable, linear regression was used to account for instable model parameters due to an excess in zeros (i.e. non-smokers), as has been applied before [24]. All models were adjusted for baseline values of the health behavior outcome of interest, age and gender, and were conducted according to the intention-to-treat principle. The interaction of the intervention with time was evaluated to test whether immediate effects differed from sustained effects. The interaction of the intervention with time was non-statistically significant for all outcomes, except for MPA. For consistency, both measurement moments were added to the mixed model in all analyses, in which a random intercept for each participant was added.

Two-stage meta-analyses were used to analyze overall intervention effects of the programs on the health behavior outcomes (dependent variable), as well as to evaluate whether the effectiveness of the health promotion programs differed by SEP group. The overall effect of SEP and the interaction term of intervention*SEP were added to statistically test for SEP differences. SEP-stratified analyses were conducted, to fulfil our a-priori aim of assessing differential effectiveness of health behavior programs across SEP groups.

Socioeconomic inequalities regarding compliance (continuous outcome, using linear regression) were analyzed. Additionally, the effectiveness of the health promotion programs was assessed in participants with low and high compliance, modelling these two groups of participants separately. These models were also stratified by SEP to assess socioeconomic inequalities in the effectiveness of the interventions in both low and high compliant groups.

For clustered trials, intra-class correlation coefficients (ICCs) were estimated to evaluate the variance within and between clusters (e.g. worksite or company level). For outcomes with ICCs> 0·10 (i.e. for MPA, VPA, MVPA and Fruit; see Supplementary file 1), a random intercept for clusters was added to the model. Heterogeneity among studies was assessed in an exclusion sensitivity analysis, in which each of the studies was subsequently left out of the analysis, assessing its impact on the overall effects. All statistical analyses were conducted using Stata (version 14). Review Manager (version 5.3.5) was used to make forest plots. For all analyses, the level of statistical significance was set at *p* < 0·05.

## Results

### Individual participant data meta-analysis

**Fig. 1:**
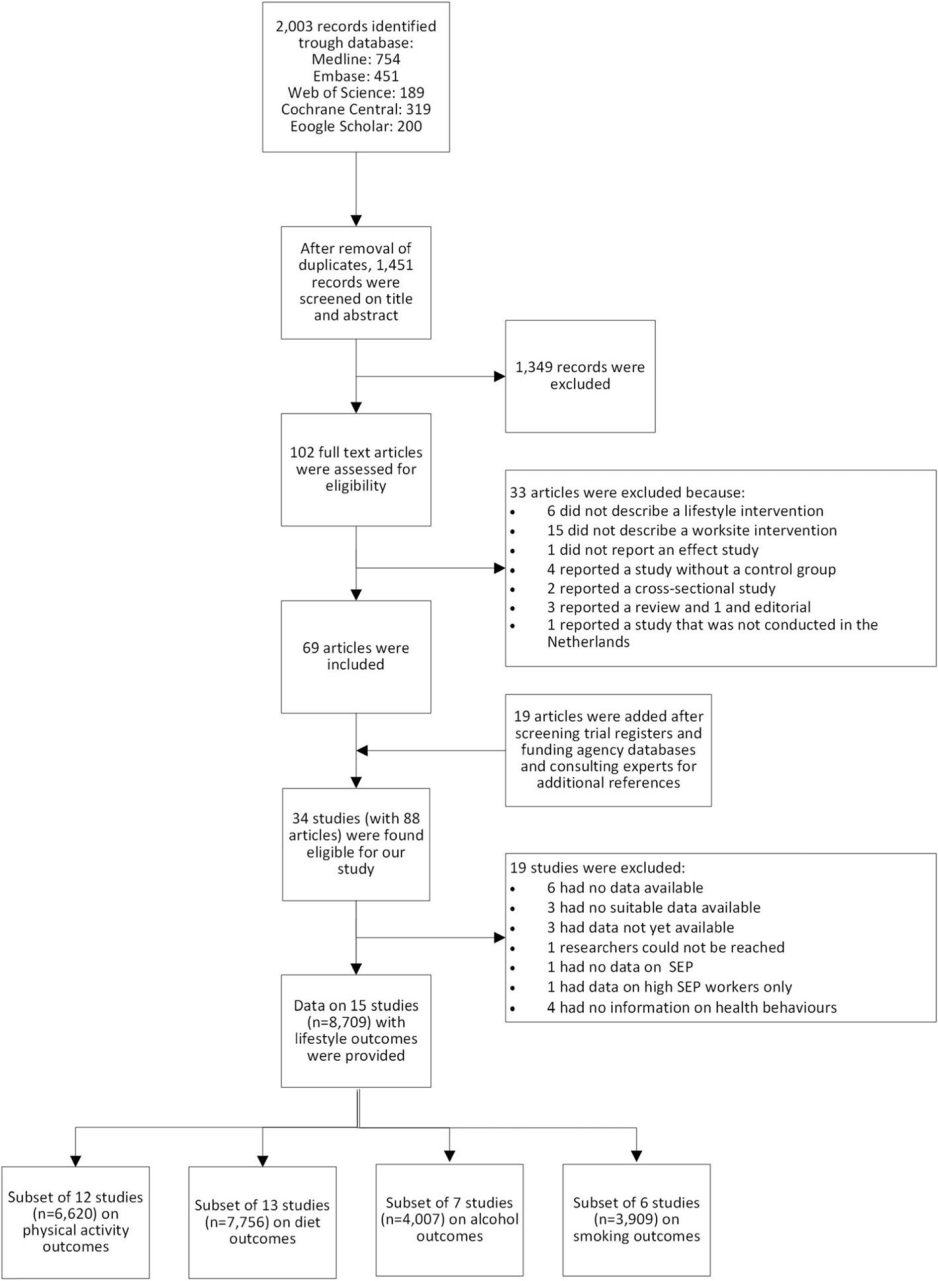
Flow chart of study inclusion process

Of the 34 original studies for which researchers were contacted (Fig. 1), 19 studies were excluded because of various reasons (Supplementary file 2): data were not available anymore (*N* = 6) or yet (*N* = 3), the researchers could not be reached (*N* = 1), data were not suitable (e.g., because they were not on an individual level, N = 3), no relevant outcome data (*N* = 4), or the study did not have information on SEP (N = 1), or described a single SEP group only (N = 1). Data of 15 studies (with *n* = 8709 participants) were included for the current analyses (Table 1, Supplementary file 3–4) [23, 25–38].

**Table 1:** Main characteristics of studies included in this meta-analysis

First author	Design	Study population characteristics					
		**N**	**Age (mean (SD))**	**% Female**	**Socioeconomic position**		
					**Low**	**Intermediate**	**High**
van Berkel [25]	RCT	257	45·5 (9·5)	67%	2%	17%	81%
Brug [26]	RCT	315	39·9 (10·1)	98%	54%	44%	2%
Coffeng [27]	C-RCT^1^	413	41·3 (10·3)	40%	2%	42%	56%
Engbers [28]	CT	531	45·4 (9·2)	40%	3%	31%	67%
Groeneveld [23]	RCT	816	46·1 (9·3)	0%	76%	24%	0%
Houkes [29]	CT	205	48·6 (9·3)	13%	10%	62%	27%
Kouwenhoven-Pasmooij [30]	C-RCT	451	50·9 (5·6)	18%	18%	53%	28%
Oenema [31]	RCT^1^	765	41·4 (9·0)	41%	20%	38%	41%
Robroek [32]	C-RCT	924	42·1 (10·1)	51%	22%	33%	45%
Steenhuis [33]	C-RCT	928	38·0 (9·8)	38%	2%	37%	60%
Strijk [34]	RCT	730	52·9 (4·9)	75%	10%	29%	61%
Verweij [35]	C-RCT	522	46·7 (8·3)	37%	12%	35%	53%
Viester [36]	RCT	314	46·6 (9·7)	0%	73%	26%	1%
van Wier [37]	RCT^1^	1320	43·4 (8·6)	34%	5%	35%	60%
Wierenga [38]	CT	218	43·1 (11·4)	78%	3%	17%	79%
Total		8709	44·3 (9·8)	40%	20%	34%	47%

C-RCT = Cluster randomized controlled trial, RCT = Randomized controlled trial, CT = (non-randomized) controlled trial

^1^The study by Coffeng et al. consisted of 3 intervention arms with different health promotion programs, the study by van Wier et al. consisted of two arms, the study by Oenema et al. consists of two control arms

### Individual participant dataset characteristics

Seven studies were randomized controlled trials (RCT) [23, 25, 26, 31, 34, 36, 37], five studies were cluster RCTs [27, 30, 32, 33, 35], and three studies were non-randomized controlled trials [28, 29, 38]. The methodological quality of one study was ‘fair’ [29], of ten studies ‘good’ [26–28, 30–34, 37, 38] and of four studies ‘excellent’ [23, 25, 35, 36] (Supplementary file 5).

Six studies included workers from all three SEP groups [24, 29, 31, 32, 34, 35]. The majority of the participants was from intermediate and high SEP groups in six studies [25, 27, 28, 33, 37, 38], with < 10% of the study sample from low SEP. In three studies the majority of the participants was from low and intermediate SEP [23, 26, 36], with < 10% of the study sample from high SEP. In four studies only participants at risk, selected based on their risk profile (e.g. with high BMI or unhealthy behavior), were included and received a selective/indicated health promotion program [23, 30, 35, 37]. The other health promotion programs were universally provided to samples of the general working populations. Most studies included both men and women, whereas two studies only included males (i.e. construction workers) [23, 36].

Health promotion programs consisted of advice, counselling, environmental components or combinations of these components, and were delivered at individual and/or group level (Supplementary file 4). Delivery was performed face-to-face, using e/mHealth or through environmental changes. In three studies, multiple health promotion programs were evaluated in a multi-armed trial [27, 33, 37], while in one study two different control groups were considered [31].

From data of 13 studies [23–25, 27, 29, 31–38], compliance was on average 51% (SD 40%). Compliance did not differ, neither for intermediate compared to low SEP workers (beta 0·06 [95%CI 4·26–4·14]), nor for high SEP compared to moderate SEP workers (beta 1·97 [95%CI -2·51 6·46]).

### Intervention effects

#### Physical activity

Twelve studies (*n* = 6620) reported the effect of health promotion programs on physical activity [23, 25, 27–30, 32, 34–38], ten studies on MPA, 11 on VPA, and 11 others on MVPA (Table 1). Overall, no statistically significant effects of the health promotion programs on physical activity were shown, nor did these effects differ across SEP group (Table 2, individual study effects in Supplementary file 6–8). Overall effect sizes ranged from beta 0·01 [95%CI − 0·04 0·06] for MPA to beta 0·03 [95%CI − 0·02 0·08] for MVPA. This corresponds to 5 [95%CI − 20 90] and 18 [95%CI 0106] minutes/week of these activities when z-scores are converted for the most commonly used measurement method.

**Table 2:** Findings regarding the effectiveness of 13 health promotion programs in 7598 workers on health behaviour outcomes. Results are shown from two-stage meta-analyses with linear mixed modelling for which we assessed intervention effects on health behaviours (physical activity, diet, alcohol use, and smoking). All models were adjusted for baseline values of the outcomes, age and gender. Total effects and those stratified by SEP are shown

	Total			Low SEP			Intermediate SEP			High SEP		
	N	n	beta [95% CI]	N	n	beta [95% CI]	N	n	beta [95% CI]	N	n	beta [95% CI]
**Physical activity**												
MPA (z-score)	10	5064	0·01 [−0·04 0·06]	8	1297	0·07 [− 0·04 0·18]	10	1687	0·01 [− 0·06 0·08]	8	2067	−0·01 [− 0·10 0·09]
VPA (z-score)	11	5798	0·01 [−0·02 0·04]	8	1300	0·01 [−0·03 0·05]	11	1728	0·01 [−0·03 0·06]	9	2248	0·05 [0·00 0·09]
MVPA (z-score)	11	6418	0·03 [−0·02 0·08]	8	1346	0·08 [0·00 0·17]	11	2068	0·04 [− 0·04 0·13]	9	2984	0·02 [−0·06 0·10]
**Dietary intake**												
Fruit (z-score)	13	7598	0·12 [0·08 0·15]	11	1614	0·20 [0·12 0·28]	13	2515	0·05 [−0·01 0·10]	10	3455	0·10 [0·06 0·15]
Vegetables (z-score)	12	7024	0·02 [−0·01 0·06]	10	1551	−0·03 [− 0·16 0·10]	12	2343	0·06 [− 0·01 0·12]	9	3112	0·02 [−0·04 0·07]
Snacks (z-score)	9	5626	−0·04 [− 0·09 0·02]	9	1337	0·04 [−0·11 0·19]	9	1951	−0·05 [− 0·11 0·01]	6	2329	−0·04 [− 0·13 0·05]
Fat (z-score)	5	3837	−0·02 [− 0·08 0·04]	5	421	0·00 [− 0·23 0·24]	5	1395	0·01 [−0·07 0·09]	4	2015	−0·05 [− 0·12 0·03]
**Alcohol (z-score)**	7	4007	0·01 [−0·03 0·05]	6	1162	0·02 [−0·08 0·12]	7	1345	0·01 [−0·05 0·08]	5	1490	0·03 [0·00 0·07]
**Smoking (dichotomous)**	6	3909	0·01 [−0·02 0·03]	5	986	0·01 [−0·04 0·06]	6	1353	−0·01 [− 0·05 0·04]	5	1563	0·00 [−0·02 0·03]

Effects sizes are expressed in betas with 95% confidence intervals (95% CI). Statistically significant intervention*SEP effects (with low SEP as reference category) are depicted with a *

Stronger effects were only found for VPA among those with high compliance than those with low compliance, (beta 0·03 [95%CI 0·00 0·06] and 0·00 [95%CI − 0·05 0·06], respectively; Table 3). A statistically significant intervention*SEP interaction effect was only found for MPA among those with high compliance, with stronger effects for those with low compared to high SEP (beta 0·17 [95%CI − 0·01 0·34] and 0·01 [95%CI − 0·09 0·11], respectively).

**Table 3:** Findings regarding the effectiveness of the health promotion programs on health behaviour outcomes, stratified by high and low compliance. Results are shown from two-stage meta-analyses with linear mixed modelling for which we assessed intervention effects on health behaviours (physical activity, diet, alcohol use, and smoking). All models were adjusted for baseline values of the outcomes, age and gender. Total effects and those stratified by SEP are shown

		Total			Low SEP			Intermediate SEP			High SEP		
	**Compliance**	**n**	**N**	**beta [95% CI]**	**n**	**N**	**beta [95% CI]**	**n**	**N**	**beta [95% CI]**	**n**	**N**	**beta [95% CI]**
**Physical activity**													
MPA (z-score)	Low	862	9	0·05 [− 0·01 0·11]	228	4	0·07 [− 0·06 0·19]	299	9	0·04 [−0·06 0·14]	324	7	0·07 [− 0·03 0·17]
	High	1118	9	0·02 [−0·03 0·07]	291	5	0·17 [−0·01 0·34]	368	8	−0·04 [− 0·14 0·06]	440	7	0·01 [−0·09 0·11]*
VPA (z-score)	Low	912	10	0·00 [− 0·05 0·06]	228	4	0·07 [− 0·12 0·26]	299	9	0·03 [−0·03 0·08]	363	8	0·05 [−0·01 0·11]
	High	1162	10	0·03 [0·00 0·06]	291	5	0·04 [0·00 0·08]	368	8	0·00 [−0·06 0·07]	474	8	0·08 [0·02 0·14]
MVPA (z-score)	Low	1318	10	0·06 [0·00 0·11]	249	5	0·07 [−0·05 0·19]	452	9	0·04 [−0·06 0·14]	597	8	0·06 [−0·01 0·14]
	High	1575	10	0·05 [0·00 0·10]	313	6	0·11 [0·00 0·22]	477	8	0·01 [−0·09 0·11]	759	8	0·06 [−0·02 0·14]
**Dietary intake**													
Fruit (z-score)	Low	1433	11	0·09 [0·04 0·14]	247	5	0·14 [0·00 0·28]	489	10	0·03 [−0·06 0·11]	673	9	0·09 [0·02 0·16]
	High	1552	11	0·13 [0·08 0·18]	296	6	0·14 [−0·04 0·31]	484	9	0·06 [−0·02 0·14]	749	9	0·13 [0·07 0·19]
Vegetables (z-score)	Low	1425	10	0·02 [−0·03 0·07]	249	5	0·07 [−0·04 0·19]	485	9	0·11 [0·02 0·20]	668	8	−0·02 [− 0·10 0·07]
	High	1441	10	0·05 [−0·01 0·11]	280	5	0·07 [−0·05 0·18]	446	8	0·07 [−0·02 0·15]	651	7	0·06 [−0·04 0·16]
Snacks (z-score)	Low	1059	7	−0·05 [− 0·10 0·00]	202	4	−0·03 [− 0·12 0·05]	390	7	− 0·05 [− 0·13 0·03]	455	5	−0·05 [− 0·15 0·06]
	High	1194	8	−0·08 [− 0·15–0·02]	255	4	−0·08 [− 0·17 0·01]	371	6	− 0·10 [− 0·18–0·02]	549	5	−0·09 [− 0·22 0·03]
Fat (z-score)	Low	732	3	−0·01 [− 0·10 0·08]	36	2	−0·18 [− 0·76 0·41]	281	3	0·01 [− 0·12 0·14]	409	3	−0·01 [− 0·13 0·10]
	High	755	3	−0·10 [− 0·20 0·00]	41	2	−0·31 [− 0·56–0·07]	264	3	−0·03 [− 0·14 0·09]	445	3	− 0·12 [− 0·21–0·02]
**Alcohol (z-score)**	Low	1082	6	0·03 [− 0·02 0·08]	211	4	−0·03 [− 0·10 0·04]	384	5	0·02 [− 0·08 0·12]	474	5	0·04 [−0·01 0·08]
	High	1069	6	0·00 [−0·04 0·04]	257	4	-0·02 [−0·09 0·05]	331	5	-0·01 [−0·08 0·07]	468	5	0·02 [−0·06 0·09]
**Smoking (dichotomous)**	Low	1067	7	0·01 [−0·02 0·04]	233	5	0·03 [−0·04 0·10]	371	6	-0·01 [−0·08 0·05]	450	5	-0·01 [−0·03 0·02]
	High	1047	7	0·00 [−0·03 0·03]	279	5	0·01 [−0·07 0·09]	313	6	-0·01 [−0·05 0·03]	442	5	0·00 [−0·02 0·02]

#### Diet

Thirteen studies (*n* = 7756) reported the effects on diet [23, 25, 26, 28, 29, 31–38] (Table 2; individual study effects in Supplementary file 9–12). Compared to the control groups, participants receiving health promotion programs reported an increase in fruit intake (beta 0·12 [95%CI 0·08 0·55]), corresponding to 0.2 [0.1 0.2] pieces of fruit/day. No statistically significant effects of the health promotion programs were found for vegetable, fat and snack intake No differences in effects were found across SEP groups.

Effects were stronger for those with high compared to low compliance to the health promotion programs, showing statistically significant more fruit and less snack intake (Table 3). No between SEP group differences were found in either the high or low compliance group.

#### Alcohol and smoking

Seven studies (*n* = 44,007) reported the effects on alcohol intake [23, 29, 30, 32, 36–38], and six (*n* = 3909) reported the effects on smoking [23, 29, 30, 32, 37, 38] (Table 2; individual study effects in Supplementary file 13–14). No effects were found on alcohol use or smoking and no differences in effects on smoking cessation and reducing alcohol intake were found between workers with low and high compliance to the health promotion programs (Table 3). None of the analyses showed SEP group differences (Table 3).

The exclusion sensitivity analysis showed that all outcomes remained stable and were independent from removing particular studies from the dataset (data not reported).

## Discussion

This meta-analysis did not show inequalities in the effectiveness of worksite health promotion programs across SEP groups (operationalized by educational level). To our knowledge, this study is the first IPD meta-analysis on workplace health promotion programs, which allowed us to address socioeconomic inequalities in these programs and to assess the role of compliance. Our lack of evidence for socioeconomic inequalities in the effectiveness of the reported health promotion programs was in line with earlier research among adults [39] and specifically for worksite health promotion programs [12]. However, overall no or small effects were found of health promotion programs on physical activity, diet, smoking and alcohol intake. These null-findings generally are in line with the modest and inconclusive findings of earlier conventional reviews on health promotion programs in occupational settings [6–9], and underline the overall ineffectiveness of such programs. This overall ineffectiveness has reduced the likelihood of finding socioeconomic inequalities in workplace health promotion program effectiveness.

Programs aimed at behavior changes, such as those based on counselling and advice, which are the majority of the interventions in this IPD meta-analysis, are more likely to be taken up by workers of higher SEP [40]. Lower SEP groups might be less likely to perceive the need for these changes, as they are hindered by more pressing struggles in their daily life, including relational, physical, emotional and/or financial issues [41]. Possibly as a result of that, programs that are focused on environmental factors could be more effective for lower SEP groups. Indeed, two earlier meta-analyses showed that (worksite) programs with environmental components showed higher absolute effectiveness in lower SEP groups, while some programs that mainly focused on behavior changes showed higher absolute effectiveness in high SEP groups [12, 42]. Only a limited number of worksite health promotion programs based on environmental components were available in the current meta-analysis and these focused on small changes in the environment, e.g. the availability of fruit at the workplace [25, 27] or signs that nudge workers to use the stairs [28]. Presumably, more drastic changes to the (economic, physical, social/cultural or political) environment are needed to introduce more substantial changes in physical activity and dietary choices [43]. In integrated programs, such elements should preferably be combined with additional effective components for low SEP workers as found in our study, such as intense (multi-session) programs [12] and messages tailored to the target population [14].

Compliance was generally low (on average 51%), which may have contributed to our null-findings. Compliance was slightly (and non-statistically significantly) lower in the low and moderate SEP groups compared to the high SEP group. Also, workers with high compliance showed statistically significantly beneficial effects for VPA, MVPA, fruit and snack intake. Although this indicates that there could be a potential for better effectiveness when compliance and sustained engagement of low SEP groups can be increased, it needs to be acknowledged that effects of the programs reported in our IPD meta-analysis will likely remain small.

### Methodological strengths and limitations

A strength of this IPD meta-analysis is that we have collected individual participant study data, which combined, has sufficient statistical power to conduct stratified analyses for SEP. In addition, our dataset enabled us to evaluate the association of compliance on program effectiveness. Moreover, we addressed publication bias by also incorporating unpublished data [29]. An additional advantage of our study is the high methodological quality of the underlying studies, with most data being from RCTs with ‘good’ or ‘excellent’ methodological quality. We only used data from Dutch studies on worksite health promotion programs. This has the advantage of being able to draw conclusions from homogeneous samples of workers within the same national context of social security and occupational health care. A limitation is, however, that extrapolating our findings to other countries should be done with due caution. Extrapolating our findings should also be done with caution since some included studies were executed some time ago, with the oldest study published in 1996 [26]. It is possible that content and way of delivery of worksite health promotion programs have changed since. Finally, the current meta-analysis was based on worksite health promotion programs. People without employment are more likely to have poorer health [44] and a lower SEP [45], than those who do have paid work. Included worksite health promotion programs do, however, not include this vulnerable group of unemployed persons and generalizing our findings to all low SEP groups should be done with caution.

A drawback of our IPD methodology is the dependency on researchers being able/willing to share their data. Of 34 identified studies, data from 19 could not be obtained. While for some studies it appeared that the study was not eligible after contacting the researchers, data were not available of nine studies and researchers of one study could not be reached. To conduct IPD meta-analyses, such as the current one, it is therefore of great importance to store study data such that they can be used by others and that data-management is transferred to another person when researchers leave from an institution. Moreover, during the harmonization procedure, concessions had to be made, which has inherently led to a loss of data detail. For example, while we aimed to do so according to our published protocol [18], due to insufficient information no distinction could be made between domains (i.e. work and leisure-time) of physical activity, while sedentary behavior could also not be assessed. Moreover, as limited or no information was available on other indicators of SEP, with only two studies reporting on income [27, 34] and one study reporting on occupational class [23], SEP was operationalized as educational level for almost all studies to harmonize data across studies according to our pre-registered protocol [18].

In contrast to what was stipulated in our protocol [18], study reach could not be analyzed as such information was not available and/or could not be harmonized across studies. Moreover, compliance was often measured subjectively in the underlying studies. Future researchers should be encouraged to describe process measures, such as reach and compliance, using a standard framework, as it might help to explain the (in)effectiveness of programs.

## Conclusion

In this first IPD meta-analysis on worksite health promotion programs, no apparent socioeconomic inequalities in program effectiveness were found (with SEP operationalized by educational level). Workplace health promotion programs were in general not effective and no socioeconomic inequalities in program compliance were found. Even though for participants with high compliance positive effects of health promotion programs were found on VPA, MVPA, fruit and snack intake, these effects remained small. Therefore, the results of the current study emphasize the need for new directions in health promotion programs to address socioeconomic health inequalities.

## Supplementary information

**Additional file 1.** Intra-class correlation (ICC) depicting the within and between variance for each of the outcome measures in studies with a cluster randomized design. ICCs> 0.10 are printed in bold.

**Additional file 2.** Excluded studies after contacting researchers.

**Additional file 3.** Included studies.

**Additional file 4.** Main characteristics of studies included in the individual participant data meta-analysis.

**Additional file 5.** Methodological quality of the studies included in the individual participant data meta-analysis.

**Additional file 6.** Forrest plot depicting the individual study effects of the health promotion programs regarding moderate physical activity. Findings are stratified by socioeconomic position: low socioeconomic position (left panel), intermediate socioeconomic position (middle panel) and high socioeconomic position (right panel).

**Additional file 7.** Forrest plot depicting the individual study effects of the health promotion programs regarding vigorous physical activity. Findings are stratified by socioeconomic position: low socioeconomic position (left panel), intermediate socioeconomic position (middle panel) and high socioeconomic position (right panel).

**Additional file 8.** Forrest plot depicting the individual study effects of the health promotion programs regarding moderate-to-vigorous physical activity. Findings are stratified by socioeconomic position: low socioeconomic position (left panel), intermediate socioeconomic position (middle panel) and high socioeconomic position (right panel).

**Additional file 9.** Forrest plot depicting the individual study effects of the health promotion programs regarding fruit intake. Findings are stratified by socioeconomic position: low socioeconomic position (left panel), intermediate socioeconomic position (middle panel) and high socioeconomic position (right panel).

**Additional file 10.** Forrest plot depicting the individual study effects of the health promotion programs regarding vegetable intake. Findings are stratified by socioeconomic position: low socioeconomic position (left panel), intermediate socioeconomic position (middle panel) and high socioeconomic position (right panel).

**Additional file 11.** Forrest plot depicting the individual study effects of the health promotion programs regarding snack intake. Findings are stratified by socioeconomic position: low socioeconomic position (left panel), intermediate socioeconomic position (middle panel) and high socioeconomic position (right panel).

**Additional file 12.** Forrest plot depicting the individual study effects of the health promotion programs regarding fat intake. Findings are stratified by socioeconomic position: low socioeconomic position (left panel), intermediate socioeconomic position (middle panel) and high socioeconomic position (right panel).

**Additional file 13.** Forrest plot depicting the individual study effects of the health promotion programs regarding alcohol intake. Findings are stratified by socioeconomic position: low socioeconomic position (left panel), intermediate socioeconomic position (middle panel) and high socioeconomic position (right panel).

**Additional file 14.** Forrest plot depicting the individual study effects of the health promotion programs regarding smoking. Findings are stratified by socioeconomic position: low socioeconomic position (left panel), intermediate socioeconomic position (middle panel) and high socioeconomic position (right panel).

## Data Availability

Aggregated data supporting the conclusions of this article are included within the article (and its additional file(s)). Individual participant data remain under ownership of the original researchers.
