# Supplementary material for: Socioeconomic inequalities in effectiveness of and compliance to workplace health promotion programs: an individual participant data (IPD) meta-analysis
Source: Int J Behav Nutr Phys Act. 2020 Sep 4;17:112. doi: 10.1186/s12966-020-01002-w (PMC7650284; doi:10.1186/s12966-020-01002-w)
Supplement: Supplementary file 1 — Additional file 1. Intra-class correlation (ICC) depicting the within and between variance for each of the outcome measures in studies with a cluster randomized design. ICCs> 0.10 are printed in bold. [file 12966_2020_1002_MOESM1_ESM.docx]

Supplementary file 1. Intra-class correlation (ICC) depicting the within and between variance for each of the outcome measures in studies with a cluster randomized design. ICCs>0.10 are printed in bold.

| First author | **MPA** | **VPA** | **MVPA** | **Smoking** | **Alcohol** | **Fruit** | **Vegetables** | **Snacks** | **Fat** |
| --- | --- | --- | --- | --- | --- | --- | --- | --- | --- |
| Coffeng^23^ | 0.08 | 0.02 | 0.07 | 0.00 | 0.00 | 0.00 | 0.00 | 0.00 | 0.00 |
| Verweij^32^ | 0.05 | 0.03 | 0.06 | 0.00 | 0.00 | 0.06 | 0.05 | 0.04 | 0.00 |
| Robroek^28^ | **0.15** | **0.11** | **0.14** | 0.09 | 0.10 | **0.11** | 0.08 | 0.00 | 0.00 |
| Kouwenhoven-Pasmooij^26^ | 0.08 | 0.03 | **0.11** | 0.05 | 0.04 | 0.00 | 0.00 | 0.00 | 0.00 |
| **Steenhuis^29^** | 0.00 | 0.00 | 0.00 | 0.00 | 0.00 | 0.01 | 0.05 | 0.03 | 0.03 |
