# Supplementary material for: Socioeconomic inequalities in effectiveness of and compliance to workplace health promotion programs: an individual participant data (IPD) meta-analysis
Source: Int J Behav Nutr Phys Act. 2020 Sep 4;17:112. doi: 10.1186/s12966-020-01002-w (PMC7650284; doi:10.1186/s12966-020-01002-w)
Supplement: Supplementary file 2 — Additional file 2. Excluded studies after contacting researchers. [file 12966_2020_1002_MOESM2_ESM.docx]

Supplementary file 2. Excluded studies after contacting researchers.

| **Study nr** |  | **Reason for exclusion** | **Study name** | **Articles** |
| --- | --- | --- | --- | --- |
| 1 | 1 | No data available |  | Proper K, A J van der Beek, V Hildebrandt, J Twisk, W van Mechelen. Worksite health promotion using individual counselling and the effectiveness on sick leave; results of a randomised controlled trial. Occup Environ Med. 2004; 61(3): 275–279.  1. Proper KI, Hildebrandt VH, Van der Beek AJ, Twisk JW, Van Mechelen W. Effect of individual counseling on physical activity fitness and health: a randomized controlled trial in a workplace setting. Am J Prev Med. 2003; 24(3): 218-26.  Proper KI, de Bruyne MC, Hildebrandt VH, van der Beek AJ, Meerding WJ, van Mechelen W. Costs, benefits and effectiveness of worksite physical activity counseling from the employer's perspective. Scand J Work Environ Health. 2004; 30(1): 36-46. |
| 2 | 2 | No data available | NHF-NRG In Balance-project | 1. Kwak L, Kremers SP, Werkman A, Visscher TL, van Baak MA, Brug J. The NHF-NRG In Balance-project: the application of Intervention Mapping in the development, implementation and evaluation of weight gain prevention at the worksite. Obes Rev. 2007; 8(4):347-361. 2. Kwak L, Kremers SP, Candel MJ, Visscher TL, Brug J, van Baak MA. Changes in skinfold thickness and waist circumference after 12 and 24 months resulting from the NHF-NRG In Balance-project. Int J Behav Nutr Phys Act. 2010; 7: 26. 3. Kwak L, Kremers SP, Visscher TL, van Baak MA, Brug J. Behavioral and cognitive effects of a worksite-based weight gain prevention program: the NHF-NRG in balance-project. J Occup Environ Med. 2009; 51(12): 1437-1446. |
| 3 | 3 | No data available | Campagne ‘lunchwandelen’ | 1. De Kraker, H. and Hendriksen, I. and , Hildebrandt, V. and De Korte, E. and Van Maas, E. D. The effect of a campaign to stimulate walking during lunch break on the physical activity behavior of employees, 2005, Geneeskd Sport |
| 4 | 4 | No data available |  | 1. Maes, S. and Kittel, F. and Scholten, H. and Verhoeven, C. Healthier Work at Brabantia', a comprehensive approach to wellness at the worksite,1992, SAF SCI, 15(4): 351-366. 2. Maes, S. and Verhoeven, C. and Kittel, F. and Scholten, H. Effects of a Dutch work-site wellness-health program: The Brabantia project,1998, Am J Public Health, 88(7): 1037-1041. |
| 5 | 5 | No data available |  | 1. Colkesen, E. B. Ferket, B. S. Tijssen, J. G. P. Kraaijenhagen, R. A. van Kalken, C. K. Peters, R. J. G. Effects on cardiovascular disease risk of a web-based health risk assessment with tailored health advice: A follow-up study,2011, Vasc Health Risk Manage, 7 (1): 67-74. |
| 6 | 6 | No data available |  | 1. Willemsen, M. C. and de Vries, H. and van Breukelen, G. and Genders, R., Long-term effectiveness of two Dutch work site smoking cessation programs, 1998, Health Educ Behav, 25(4): 418-435. 2. Willemsen, M. C. and Vries, H.Evaluation of a smoking cessation intervention for Dutch employees consisting of self help methods and a group programme,1995, Tobacco control, 4(4): 351-364. |
| 7 | 1 | No suitable data (no individual participant data) |  | 1. Vermeer WM, Leeuwis FH, Koprulu S, Zouitni O, Seidell JC, Steenhuis IH. The process evaluation of two interventions aimed at portion size in worksite cafeterias. J Hum Nutr Diet. 2012; 25(2): 180-188. 2. Vermeer WM, Steenhuis IH, Leeuwis FH, Heymans MW, Seidell JC. Small portion sizes in worksite cafeterias: do they help consumers to reduce their food intake? Int J Obes. 2011; 35(9): 1200-1207. 3. Vermeer WM, Alting E, Steenhuis IH, Seidell JC. Value for money or making the healthy choice: the impact of proportional pricing on consumers' portion size choices. Eur J Public Health. 2010; 20(1): 65-69. |
| 8 | 2 | No suitable data | PAM COACH | 1. Slootmaker SM, Chinapaw MJ, Schuit AJ, Seidell JC, Van Mechelen W. Feasibility and effectiveness of online physical activity advice based on a personal activity monitor: randomized controlled trial. J Med Internet Res. 2009 ;11(3):e27.. 2. Slootmaker SM, Chin A Paw MJ, Schuit AJ, Seidell JC, van Mechelen W. Promoting physical activity using an activity monitor and a tailored web-based advice: design of a randomized controlled trial [ISRCTN93896459]. BMC Public Health. 2005; 5: 134. |
| 9 | 3 | No suitable data (no individual participant data) |  | 1. Brug, J. and Steenhuis, I. and Van Assema, P. and De Vries, H. The impact of a computer-tailored nutrition intervention,1996, Preventive Medicine, 25(3): 236-242. 2. Brug, J. Dutch research into the development and impact of computer-tailored nutrition education,1999, Eur J Clin Nutr, 53: S78-S82. |
| 10 | 1 | Data not yet available (study in progress) | Continuous Abstinence Through Corporate Healthcare (CATCH) study | 1. Brandt van den F.A., Nagelhout G.E., Winkens B., Evers S.M., Kotz D., Chavannes N.H., van Schayck, C.P. The effect of financial incentives on top of behavioral support on quit rates in tobacco smoking employees: study protocol of a cluster-randomized trial. BMC Public Health, 2016; 16(1)1056, |
| 11 | 2 | Data not yet available (study in progress) |  | 1. Laan E.K., Kraaijenhagen, R. A. Peek, N. Busschers W.B., Deutekom M., Bossuyt P.M., Stronks K., Essink-Bot M.L. Effectiveness of a web-based health risk assessment with individually-tailored feedback on lifestyle behaviour: study protocol,2012, BMC Public Health, 12: 200 |
| 12 | 3 | Data not yet available (study in progress) |  | 1. Velema E, Vyth EL, Hoekstra T, Steenhuis IHM. Nudging and social marketing techniques encourage employees to make healthier food choices: a randomized controlled trial in 30 worksite cafeterias in The Netherlands. Am J Clin Nutr. 2018; 107(2):236-246. 2. Velema, E. and Vyth, E. L. and Steenhuis, I. H., Using nudging and social marketing techniques to create healthy worksite cafeterias in the Netherlands: intervention development and study design,2017, BMC Public Health, 17(1): 63, |
| 13 | 1 | Researchers could not be reached |  | 1. Valk de, R. H. [Dietary intervention in occupational medicine] Dutch,Ned Tijdschr Geneeskd, 134(7): 338-341. |
| 14 | 1 | No data on SEP |  | 1. Hendriksen, I. J. M. and Zuiderveld, B. and Kemper, H. C. G. and Bezemer, P. D., Effect of commuter cycling on physical performance of male and female employees,2000, Med Sci Sports Exerc, 32 (2): 504-510. |
| 15 | 1 | Only data on high SEP | MORE Energy mHealth intervention | 1. Drongelen van A., Beek A.J., Hlobil H., Smid T., Boot C.R. Development and evaluation of an intervention aiming to reduce fatigue in airline pilots: design of a randomised controlled trial. BMC Public Health, 2013 13: 776. 2. Drongelen van A., Boot C.R., Hlobil H., Twisk J.W., Smid T., van der Beek, A.J. Evaluation of an mHealth intervention aiming to improve health-related behavior and sleep and reduce fatigue among airline pilots. Scand J Work Environ Health, 2014; 40(6): 557-568 |
| 16 | 1 | No relevant outcomes |  | 1. Vyth, E. L. and Steenhuis, I. H. M. and Heymans, M. W. and Roodenburg, A. J. C. and Brug, J. and Seidell, J. C., Influence of Placement of a Nutrition Logo on Cafeteria Menu Items on Lunchtime Food Choices at Dutch Work Sites,2011, J Am Diet Assoc, 111(1): 131-136. |
| 17 | 2 | No relevant outcomes | Mental Vitality @ Work | 1. Gärtner FR, Nieuwenhuijsen K, Ketelaar SM, van Dijk FJ, Sluiter JK. The mental vitality @ work study: effectiveness of a mental module for workers' health surveillance for nurses and allied health care professionals on their help-seeking behavior. [J Occup Environ Med.](https://www.ncbi.nlm.nih.gov/pubmed/24064780) 2013; 55(10):1219-1229. |
| 18 | 3 | No relevant outcomes |  | 1. Niessen, M. A. J. and Kraaijenhagen, R. A. and Dijkgraaf, M. G. W. and Van Pelt, D. and Van Kalken, C. K. and Peek, N. Impact of a web-based worksite health promotion program on absenteeism,2012, J Occup Environ Med, 54(4): 404-408 |
| 19 | 4 | No relevant outcomes |  | 1. Vries de J.D., van Hooff M.L., Geurts S.A., Kompier M.A. Efficacy of an exercise intervention for employees with work-related fatigue: study protocol of a two-arm randomized controlled trial BMC Public Health, 2015; 15,1117. 2. Vries de, J. D. Van Hooff, M. L. M. Geurts, S. A. E. Kompier, M. A. J. Exercise to reduce work-related fatigue among employees: A randomized controlled trial. Scand J Work Environ Health, 2017; 43 (4): 337-349. |
