# Supplementary material for: Socioeconomic inequalities in effectiveness of and compliance to workplace health promotion programs: an individual participant data (IPD) meta-analysis
Source: Int J Behav Nutr Phys Act. 2020 Sep 4;17:112. doi: 10.1186/s12966-020-01002-w (PMC7650284; doi:10.1186/s12966-020-01002-w)
Supplement: Supplementary file 3 — Additional file 3. Included studies. [file 12966_2020_1002_MOESM3_ESM.docx]

Supplementary file 3. Included studies.

| **Study nr** | **Study name** | **Articles** |
| --- | --- | --- |
| 1 | Vitality in practice | 1. van Dongen JM, van Berkel J, Boot CR, Bosmans JE, Proper KI, Bongers PM, van der Beek AJ, van Tulder MW, van Wier MF. Long-Term Cost-Effectiveness and Return-on-Investment of a Mindfulness-Based Worksite Intervention: Results of a Randomized Controlled Trial. J Occup Environ Med. 2016; 58(6):550-60. 2. van Berkel J, Boot CR, Proper KI, Bongers PM, van der Beek AJ. Effectiveness of a worksite mindfulness-related multi-component health promotion intervention on work engagement and mental health: results of a randomized controlled trial. PLoS One. 2014; 9(1):e84118. 3. van Berkel J, Boot CR, Proper KI, Bongers PM, van der Beek AJ. Effectiveness of a worksite mindfulness-based multi-component intervention on lifestyle behaviors. Int J Behav Nutr Phys Act. 2014; 11:9. 4. van Berkel J, Boot CR, Proper KI, Bongers PM, van der Beek AJ. Process evaluation of a workplace health promotion intervention aimed at improving work engagement and energy balance. J Occup Environ Med. 2013;55(1):19-26. 5. van Berkel J, Proper KI, Boot CR, Bongers PM, van der Beek AJ. Mindful Vitality in Practice : an intervention to improve the work engagement and energy balance among workers; the development and design of the randomised controlled trial. BMC Public Health. 2011;11:736. |
| 2 | VIP in construction | 1. Viester L, Verhagen EALM, Bongers PM, van der Beek AJ. Effectiveness of a Worksite Intervention for Male Construction Workers on Dietary and Physical Activity Behaviors, Body Mass Index, and Health Outcomes: Results of a Randomized Controlled Trial. Am J Health Promot. 2018;32(3):795-805. 2. Viester L, Verhagen EA, Bongers PM, van der Beek AJ. The effect of a health promotion intervention for construction workers on work-related outcomes: results from a randomized controlled trial. Int Arch Occup Environ Health. 2015;88(6):789-98. 3. Viester L, Verhagen EA, Bongers PM, van der Beek AJ. Process evaluation of a multifaceted health program aiming to improve physical activity levels and dietary patterns among construction workers. J Occup Environ Med. 2014;56(11):1210-7. 4. Viester L, Verhagen EA, Proper KI, van Dongen JM, Bongers PM, van der Beek AJ. VIP in construction: systematic development and evaluation of a multifaceted health programme aiming to improve physical activity levels and dietary patterns among construction workers. BMC Public Health. 2012;12:89. |
| 3 | Be Active & Relax Vitality in Practice (VIP) | 1. van Dongen JM, Coffeng JK, van Wier MF, Boot CRL, Hendriksen IJM, van Mechelen W, Bongers PM, van der Beek AJ, Bosmans JE, van Tulder MW. The cost-effectiveness and return-on-investment of a combined social and physical environmental intervention in office employees. Health Educ Res. 2017;32(5):384-398. 2. Formanoy MA, Dusseldorp E, Coffeng JK, Van Mechelen I, Boot CR, Hendriksen IJ, Tak EC. Physical activity and relaxation in the work setting to reduce the need for recovery: what works for whom? BMC Public Health. 2016;16(1):866. 3. Coffeng JK, Boot CR, Duijts SF, Twisk JW, van Mechelen W, Hendriksen IJ. Effectiveness of a worksite social & physical environment intervention on need for recovery, physical activity and relaxation; results of a randomized controlled trial. PLoS One. 2014;9(12):e114860. 4. Coffeng JK, Hendriksen IJ, Duijts SF, Twisk JW, van Mechelen W, Boot CR. Effectiveness of a combined social and physical environmental intervention on presenteeism, absenteeism, work performance, and work engagement in office employees. J Occup Environ Med. 2014; 56(3):258-65. 5. Coffeng JK, Hendriksen IJ, van Mechelen W, Boot CR. [Process evaluation of a worksite social and physical environmental intervention.](https://www.ncbi.nlm.nih.gov/pubmed/24270291) J Occup Environ Med. 2013; 55(12):1409-20. 6. Coffeng JK, Hendriksen IJ, Duijts SF, Proper KI, van Mechelen W, Boot CR. The development of the Be Active & Relax Vitality in Practice (VIP) project and design of an RCT to reduce the need for recovery in office employees. BMC Public Health. 2012;12:592. 7. Koopmans L., Coffeng J.K., Bernaards C.M., Boot C.R., Hildebrandt V.H., de Vet H.C., van der Beek A.J. Responsiveness of the individual work performance questionnaire BMC Public Health, 2014; 14: 513, |
| 4 | Balance @ work | 1. Verweij LM, Proper KI, Weel AN, Hulshof CT, van Mechelen W. Long-term effects of an occupational health guideline on employees' body weight-related outcomes, cardiovascular disease risk factors, and quality of life: results from a randomized controlled trial. Scand J Work Environ Health. 2013 1;39(3):284-94. 2. Verweij LM, Proper KI, Hulshof CT, van Mechelen W. Process evaluation of an occupational health guideline aimed at preventing weight gain among employees. J Occup Environ Med. 2011;53(7):722-9. 3. Verweij LM, Proper KI, Weel AN, Hulshof CT, van Mechelen W. The application of an occupational health guideline reduces sedentary behaviour and increases fruit intake at work: results from an RCT. Occup Environ Med. 2012;69(7):500-7. 4. Verweij LM, Proper KI, Weel AN, Hulshof CT, van Mechelen W. Design of the Balance@Work project: systematic development, evaluation and implementation of an occupational health guideline aimed at the prevention of weight gain among employees. BMC Public Health. 2009 14;9:461. 5. van Wier MF, Verweij LM, Proper KI, Hulshof CT, van Tulder MW, van Mechelen W. Economic evaluation of an occupational health care guideline for prevention of weight gain among employees. J Occup Environ Med. 2013;55(9):1100-9. 6. Verweij LM, Proper KI, Leffelaar ER, Weel AN, Nauta AP, Hulshof CT, van Mechelen W. Barriers and facilitators to implementation of an occupational health guideline aimed at preventing weight gain among employees in the Netherlands. J Occup Environ Med. 2012;54(8):954-60. 7. Verweij L.M., Proper K.I., Hulshof T.J., van Mechelen W. Appropriate implementation of a weight gain prevention guideline results in greater weight loss Obes Rev, 2011; 12: 271. 8. Wier van M., Verweij L., Proper K., Hulshof, C., Van Tulder M., van Mechelen, W., Economic evaluation of an occupational health guideline for prevention of weight gain among employees. J Sci Med Sport, 2012; 15: S238, |
| 5 | ALIFE@Work | 1. Gussenhoven AH, van Wier MF, Bosmans JE, Dekkers JC, van Mechelen W. Cost-effectiveness of a distance lifestyle counselling programme among overweight employees from a company perspective, ALIFE@Work: a randomized controlled trial. Work. 2013 1;46(3):337-46. 2. van Wier MF, Dekkers JC, Bosmans JE, Heymans MW, Hendriksen IJ, Pronk NP, van Mechelen W, van Tulder MW. Economic evaluation of a weight control program with e-mail and telephone counseling among overweight employees: a randomized controlled trial. Int J Behav Nutr Phys Act. 2012 ;9:112. 3. van Wier MF, Dekkers JC, Hendriksen IJ, Heymans MW, Ariëns GA, Pronk NP, Smid T, van Mechelen W. [Effectiveness of phone and e-mail lifestyle counseling for long term weight control among overweight employees.](https://www.ncbi.nlm.nih.gov/pubmed/21654441) J Occup Environ Med. 2011;53(6):680-6. 4. Dekkers JC, van Wier MF, Ariëns GA, Hendriksen IJ, Pronk NP, Smid T, van Mechelen W. Comparative effectiveness of lifestyle interventions on cardiovascular risk factors among a Dutch overweight working population: a randomized controlled trial. BMC Public Health. 2011 ;11(1):49. 5. van Wier MF, Ariëns GA, Dekkers JC, Hendriksen IJ, Smid T, van Mechelen W. Phone and e-mail counselling are effective for weight management in an overweight working population: a randomized controlled trial. BMC Public Health. 2009 ;9:6. 6. van Wier MF, Ariëns GA, Dekkers JC, Hendriksen IJ, Pronk NP, Smid T, van Mechelen W. ALIFE@Work: a randomised controlled trial of a distance counselling lifestyle programme for weight control among an overweight working population [ISRCTN04265725]. BMC Public Health. 2006; 6:140. |
| 6 | FoodSteps | 1. Engbers LH, van Poppel MN, van Mechelen W. [Modest effects of a controlled worksite environmental intervention on cardiovascular risk in office workers.](https://www.ncbi.nlm.nih.gov/pubmed/17187852) Prev Med. 2007; 44(4): 356-362. 2. Engbers LH, van Poppel MN, Chin A Paw M, van Mechelen W. [The effects of a controlled worksite environmental intervention on determinants of dietary behavior and self-reported fruit, vegetable and fat intake.](https://www.ncbi.nlm.nih.gov/pubmed/17044935) BMC Public Health. 2006 ;6:253. |
| 7 | Vital@Work | 1. van Dongen JM, Strijk JE, Proper KI, van Wier MF, van Mechelen W, van Tulder MW, van der Beek AJ. [A cost-effectiveness and return-on-investment analysis of a worksite vitality intervention among older hospital workers: results of a randomized controlled trial.](https://www.ncbi.nlm.nih.gov/pubmed/23439274) J Occup Environ Med. 2013;55(3):337-46. 2. Strijk JE, Proper KI, van der Beek AJ, van Mechelen W. [A worksite vitality intervention to improve older workers' lifestyle and vitality-related outcomes: results of a randomised controlled trial.](https://www.ncbi.nlm.nih.gov/pubmed/22268128) J Epidemiol Community Health. 2012; 66(11):1071-1078. 3. Strijk JE, Proper KI, van der Beek AJ, van Mechelen W. [A process evaluation of a worksite vitality intervention among ageing hospital workers.](https://www.ncbi.nlm.nih.gov/pubmed/21663610) Int J Behav Nutr Phys Act. 2011; 8: 58. 4. Strijk JE, Proper KI, van Mechelen W, van der Beek AJ. [Effectiveness of a worksite lifestyle intervention on vitality, work engagement, productivity, and sick leave: results of a randomized controlled trial.](https://www.ncbi.nlm.nih.gov/pubmed/22740100) Scand J Work Environ Health. 2013; 39(1): 66-75. 5. Strijk JE, Proper KI, van der Beek AJ, van Mechelen W. [The Vital@Work Study. The systematic development of a lifestyle intervention to improve older workers' vitality and the design of a randomised controlled trial evaluating this intervention.](https://www.ncbi.nlm.nih.gov/pubmed/19903345) BMC Public Health. 2009; 9: 408. 6. Strijk J. Proper K. van Mechelen W. van der Beek A. A process evaluation of a vitality intervention among older hospital workers Occup Environ Med, 2011; 1(1): A124, |
| 8 | Health under construction study | 1. Groeneveld IF, Proper KI, van der Beek AJ, Hildebrandt VH, van Mechelen W. [Short and long term effects of a lifestyle intervention for construction workers at risk for cardiovascular disease: a randomized controlled trial.](https://www.ncbi.nlm.nih.gov/pubmed/22040007) BMC Public Health. 2011; 11: 836. 2. Groeneveld IF, Proper KI, Absalah S, van der Beek AJ, van Mechelen W. [An individually based lifestyle intervention for workers at risk for cardiovascular disease: a process evaluation.](https://www.ncbi.nlm.nih.gov/pubmed/21721966) Am J Health Promot. 2011;25(6):396-401. 3. Groeneveld IF, van Wier MF, Proper KI, Bosmans JE, van Mechelen W, van der Beek AJ. [Cost-effectiveness and cost-benefit of a lifestyle intervention for workers in the construction industry at risk for cardiovascular disease.](https://www.ncbi.nlm.nih.gov/pubmed/21654430) J Occup Environ Med. 2011;53(6):610-617. 4. Groeneveld IF, Proper KI, van der Beek AJ, van Mechelen W. [Sustained body weight reduction by an individual-based lifestyle intervention for workers in the construction industry at risk for cardiovascular disease: results of a randomized controlled trial.](https://www.ncbi.nlm.nih.gov/pubmed/20692282) Prev Med. 2010; 51(3-4):240-246. 5. Groeneveld IF, Proper KI, van der Beek AJ, Hildebrandt VH, van Mechelen W. [Factors associated with non-participation and drop-out in a lifestyle intervention for workers with an elevated risk of cardiovascular disease.](https://www.ncbi.nlm.nih.gov/pubmed/19951417) Int J Behav Nutr Phys Act. 2009; 6:80. 6. Groeneveld IF, Proper KI, van der Beek AJ, van Duivenbooden C, van Mechelen W. [Design of a RCT evaluating the (cost-) effectiveness of a lifestyle intervention for male construction workers at risk for cardiovascular disease: the health under construction study.](https://www.ncbi.nlm.nih.gov/pubmed/18173844) BMC Public Health. 2008 3;8:1. |
| 9 |  | 1. Robroek SJ, Bredt FJ, Burdorf A. The (cost-)effectiveness of an individually tailored long-term worksite health promotion programme on physical activity and nutrition: design of a pragmatic cluster randomised controlled trial. BMC Public Health. 2007; 7: 259. 2. Robroek SJ, Lindeboom DE, Burdorf A, Initial and Sustained Participation in an Internet-delivered Long-term Worksite Health Promotion Program on Physical Activity and Nutrition J Med Internet Res. 2012; 14(2): e43. 3. Robroek SJ, Polinder S, Bredt FJ, Burdorf A. Cost-effectiveness of a long-term Internet-delivered worksite health promotion programme on physical activity and nutrition: a cluster randomized controlled trial. Health Educ Res. 2012;27(3): 399-410. |
| 10 | Perfectfit | 1. Kouwenhoven-Pasmooij T.A., Djikanovic B., Robroek S.J., Helmhout P., Burdorf A., Hunink M. Design and baseline characteristics of the PerfectFit study: a multicenter cluster-randomized trial of a lifestyle intervention in employees with increased cardiovascular risk. BMC Public Health, 2015; 15: 715. 2. Kouwenhoven-Pasmooij TA, Robroek SJ, Nieboer D, Helmhout PH, Wery MF, Hunink M, Burdorf A. Quality of motivational interviewing matters: the effect on participation in health-promotion activities in a cluster randomized controlled trial. Scand J Work Environ Health. 2018, doi: 10.5271/sjweh.3716. |
| 11 | BRAVO@Work | 1. Wierenga, D. and , Engbers and Van, E. and Van, M. A worksite intervention to promote a healthy lifestyle among employees: A process evaluation,2013, Occup Environ Med, 70. 2. Wierenga, D. and Engbers, L. H. and van Empelen, P. and , Hildebrandt, V. H. and van Mechelen, W., The design of a real-time formative evaluation of the implementation process of lifestyle interventions at two worksites using a 7-step strategy (BRAVO@Work),2012, BMC Public Health, 12: 619. 3. Wierenga, D. and Engbers, L. H. and Van Empelen, P. and De Moes, K. J. and Wittink, H. and Grndemann, R. and Van Mechelen, W., The implementation of multiple lifestyle interventions in two organizations A Process Evaluation,2014, J Occup Environ Med, 56(11): 1195-1206. |
| 12 |  | 1. Brug J, Steenhuis I, van Assema P, Glanz K, De Vries H. Computer-tailored nutrition education: differences between two interventions. 1999. Health Education Research. 14(2):249-56. |
| 13 |  | 1. Steenhuis, I. and Van Assema, P. and Van Breukelen, G. and Glanz, K. and Kok, G. and De Vries, H. The impact of educational and environmental interventions in Dutch worksite cafeterias,2004, Health Promot Int, 19(3): 335-43. |
| 14 |  | 1. Oenema A, F. Tan, J. Brug. Short-term efficacy of a web-based computer-tailored nutrition intervention: main effects and mediators, 2005, Ann Behav Med, 29: 54-63. |
| 15 | Vitality @ DSM | 1. Houkes I. Vitality@DSM: participatie en effectiviteit. Een diversiteitsspecifieke evaluatie. |
