# Supplementary material for: Socioeconomic inequalities in effectiveness of and compliance to workplace health promotion programs: an individual participant data (IPD) meta-analysis
Source: Int J Behav Nutr Phys Act. 2020 Sep 4;17:112. doi: 10.1186/s12966-020-01002-w (PMC7650284; doi:10.1186/s12966-020-01002-w)
Supplement: Supplementary file 4 — Additional file 4. Main characteristics of studies included in the individual participant data meta-analysis. [file 12966_2020_1002_MOESM4_ESM.docx]

Supplementary file 4. Main characteristics of studies included in the individual participant data meta-analysis.

|  | **Health promotion program component** | | | | | **Outcomes** | | | | | | | | |
| --- | --- | --- | --- | --- | --- | --- | --- | --- | --- | --- | --- | --- | --- | --- |
| **First author** | **Intervention characteristics** | | | | **Control condition** | **Physical activity** | | | | **Diet** | | | **Alcohol^2^** | **Smoking** |
|  | **Type** | **Component** | **Level** | **Delivery** |  | **Method** | **MPA** | **VPA** | **MVPA** | **Method fruit & vegetables** | **Method fat** | **Method snack** |  |  |
| van Berkel^21^ | Universal | combined | combined | face-to-face &  e/m-Health &  environment | **generic information** | Squash | * | * | * | SFVQ |  |  |  |  |
| Brug^22^ | Universal | Advice | individual | e/m-Health | **personalized letter with tailored feedback** |  |  |  |  | Self-con. | Fat list | Fat list |  |  |
| Coffeng^23^ | Universal | 1: group counselling  2: physical environment  3: group counselling/physical environment | 1: group  2: group  3: group | 1: face-to-face  2: environment  3: face-to-face & environment | **no intervention** | Squash | * | * | * |  |  |  |  |  |
| Engbers^24^ | Universal | environment | group | environment | **no intervention** | Squash | * | * | * | SFVQ | Fat list | Fat list |  |  |
| Groeneveld^19^ | Selective/indicated | counselling | individual | face-to-face | **generic information** | Squash | * | * | * | Self-con. |  | Self-con. | * | * |
| Houkes^25^ | Universal | counselling | individual | face-to-face | **no intervention** | Self-con. | * | * |  | Self-con. |  | Self-con. | * | * |
| Kouwenhoven- Pasmooij^26^ | Selective/indicated | counselling | individual | face-to-face  e/m-Health | **generic information and personalized letter with feedback** | Self-con. | * | * | * |  |  |  | * | * |
| **Oenema^27^** | Universal | advice | Individual | e/-m health | **1: no intervention**  **2: generic information** |  |  |  |  | SFVQ | Fat list | Fat list |  |  |
| Robroek^28^ | Universal | counselling | individual | face-to-face &  e/m-Health | **standard behaviour intervention program** | IPAQ | * | * | * | SFVQ |  |  | * | * |
| **Steenhuis^29^** | Universal | 1: advice  2: more healthy food available in canteens  3: labelling of healthy food in canteens | 1: individual  2: group  3: group | 1. face-to-face  2: environment  3: environment | **no intervention** |  |  |  |  | SFVQ | Fat list | Fat list |  |  |
| Strijk^30^ | Universal | **combined** | combined | face-to-face &  environment | **no intervention** | Squash | * | * | * | SFVQ |  |  |  |  |
| Verweij^32^ | Selective/indicated | combined | Individual | face-to-face | **health appraisal and advice** | Squash | * | * | * | SFVQ |  | Fat list |  |  |
| Viester^33^ | Universal | counselling | Individual | face-to-face | **usual care** | Squash | * | * | * | Self-con. |  | Self-con. | * |  |
| van Wier^31^ | Selective/indicated | 1: counselling  2: counselling | 1: individual  2: individual | 1: face-to-face  2: e/m-Health | **generic information** | Squash |  |  | * | SFVQ | Fat list | Fat list | * | * |
| Wierenga^34^ | Universal | combined | combined | face-to-face &  environment | **usual care** | Trend |  | * | * | Self-con. |  |  | * | * |
| MPA=Moderate physical activity, VPA=Vigorous physical activity, MVPA=Moderate-to-vigorous physical activity; Self-con. = Self constructed; Trend = Trend report; SFVQ = short fruit and vegetable questionnaire  ^1^The study by Coffeng et al. consisted of 3 intervention arms with different health promotion programs, the study by van Wier et al. consisted of two intervention arms, the study by Oenema et al. consists of two control arms  ^2^All questionnaires used for alcohol and smoking were self-constructed questionnaires. | | | | | | | | | | | | | | |
