# Supplementary material for: Socioeconomic inequalities in effectiveness of and compliance to workplace health promotion programs: an individual participant data (IPD) meta-analysis
Source: Int J Behav Nutr Phys Act. 2020 Sep 4;17:112. doi: 10.1186/s12966-020-01002-w (PMC7650284; doi:10.1186/s12966-020-01002-w)
Supplement: Supplementary file 5 — Additional file 5. Methodological quality of the studies included in the individual participant data meta-analysis. [file 12966_2020_1002_MOESM5_ESM.docx]

Supplementary file 5. Methodological quality of the studies included in the individual participant data meta-analysis.

| **First author** | **Randomization** | **Blinding** | **Similarity** | **Compliance** | **Loss to follow-up** | **Intention-to-treat** | **Controlled for confounding** | **Data collection** | **Follow-up duration** | **Sum score** |
| --- | --- | --- | --- | --- | --- | --- | --- | --- | --- | --- |
| van Berkel^21^ | + | - | + | + | + | + | + | + | + | 8 (excellent) |
| Brug^22^ | + | ? | + | + | + | + | + | + | - | 7 (good) |
| Coffeng^23^ | + | - | + | - | + | + | + | + | + | 7 (good) |
| Engbers^24^ | - | - | + | ? | + | + | + | + | + | 6 (good) |
| Groeneveld^19^ | + | - | + | + | + | + | + | + | + | 8 (excellent) |
| Houkes^25^ | - | - | + | - | - | + | + | - | + | 4 (fair) |
| Kouwenhoven- Pasmooij^26^ | + | **-** | + | + | **-** | + | + | + | + | 7 (good) |
| **Oenema^27^** | + | ? | + | + | - | + | + | + | - | 6 (good) |
| Robroek^28^ | + | + | - | - | - | + | + | + | + | 6 (good) |
| **Steenhuis^29^** | + | + | - | - | - | + | + | + | + | 6 (good) |
| Strijk^30^ | + | - | + | + | - | + | + | + | + | 7 (good) |
| Verweij^32^ | + | + | + | + | + | + | + | + | + | 9 (excellent) |
| Viester^33^ | + | - | + | + | + | + | + | + | + | 8 (excellent) |
| van Wier^31^ | + | - | + | - | - | + | + | + | + | 6 (good) |
| Wierenga^34^ | - | - | + | - | - | + | + | + | + | 5 (good) |
