# Supplementary material for: Socioeconomic inequalities in effectiveness of and compliance to workplace health promotion programs: an individual participant data (IPD) meta-analysis
Source: Int J Behav Nutr Phys Act. 2020 Sep 4;17:112. doi: 10.1186/s12966-020-01002-w (PMC7650284; doi:10.1186/s12966-020-01002-w)
Supplement: Supplementary file 8 — Additional file 8. Forrest plot depicting the individual study effects of the health promotion programs regarding moderate-to-vigorous physical activity. Findings are stratified by socioeconomic position: low socioeconomic position (left panel), intermediate socioeconomic position (middle panel) and high socioeconomic position (right panel). [file 12966_2020_1002_MOESM8_ESM.docx]

Supplementary file 8. Forrest plot depicting the individual study effects of the health promotion programs regarding moderate-to-vigorous physical activity. Findings are stratified by socioeconomic position: low socioeconomic position (left panel), intermediate socioeconomic position (middle panel) and high socioeconomic position (right panel).

**
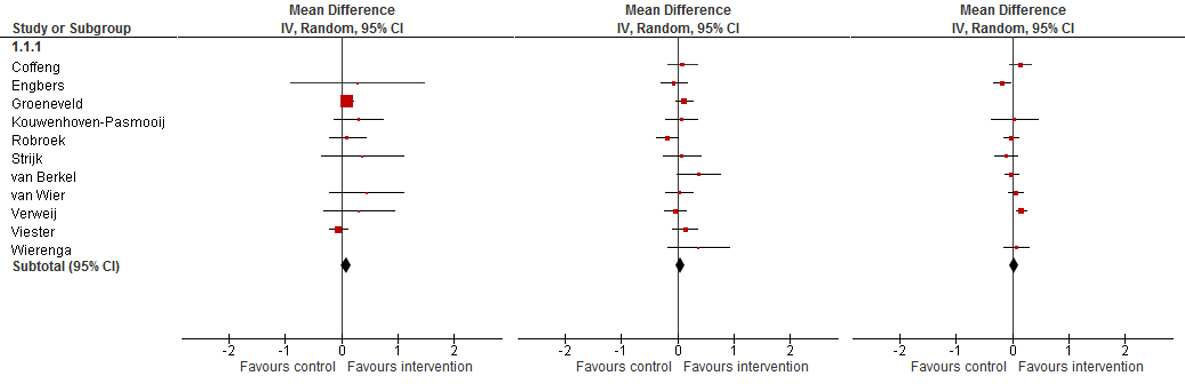
**
